# Neuroborreliosis in South Wales

**Published:** 1992-12

**Authors:** Keith KT Lim, Brendan McLean, Chris J. Burns-Cox

**Affiliations:** Registrar Department of Medicine Frenchay Hospital, Bristol; Consultant Neurologist Trelisk Hospital, Cornwall; Consultant Physician, Frenchay Hospital, Bristol


### Neuroborreliosis in South Wales

**Dr. Keith KT Lim, M.R.C.P.***
Registrar Department of Medicine
Frenchay Hospital, Bristol
**Dr. Brendan McLean, M.D., M.R.C.P.**
Consultant Neurologist
Trelisk Hospital, Cornwall
**Dr. Chris J. Burns-Cox, F.R.C.P.**
Consultant Physician,
Frenchay Hospital, Bristol
*Author to whom correspondence should be addressed.

On 23rd of March 1991 a 43 year old aircraft inspector from Chepstow was referred with six weeks increasing frontal headache, keeping him awake at night and relieved by standing up. There was no nausea,vomiting or photophobia. Five days before admission he was watching television and suddenly developed double vision more marked to the left. There was no nausea vomiting, giddiness, fevers, sweats or systemic upset. He was perfectly well prior to this. He did suffer from a recurrent rash on his thighs, trunk and limbs which had been diagnosed as granuloma annulare by a dermatologist. There was no arthropathy. He had taken up hill walking recently and visited the Forest of Dean and areas around South Wales. On examination the only positive findings were bilateral lateral recti palsy more marked on the left.

### INVESTIGATIONS

The haemoglobin was 16.2 gm/dl, the white cell count was $7.8x10^9$ /l and the platelet count was $397x10^9$ /l.The urea, electrolytes and liver function tests were normal. The plasma glucose was 5.2 mmol/l. The plasma viscosity was 1.93cp. Lumbar puncture yielded clear and colourless fluid with normal pressure. Microscopy yielded 31 lymphocytes on high power field.The CSF sugar was 3.5mmol/l and protein was 0.44 gm/l. The VDRL and TPHA serology were negative.Routine viral screen for coxackie virus, toxoplasmosis, cmv, ,brucellosis, psittacosis, and herpes simplex virus did not show rising titres.

Computed tomography (CT) with contrast and magnetic resonance imaging of the head were normal. Left carotid and vetebral angiograms were normal. The Chest X-ray and echocardiography were normal. The Borrelia burdoferi IgG was positive at 1:40.(Elisa)

A diagnosis of neuroborreliosis (Lyme Disease) was suspected and he was started on oral Amoxycillin 1 gm tds for two weeks. There was a remarkable clinical response after the first two days and the headache disappeared.The diplopia resolved completely. Repeat serology showed IgG 1:36. The western blot confirmed a specific band for Borrelia burdoferi which began to fade on repeat after treatment. The CSF specimen before treatment was not sent for Borrelia antibodies but the subsequent CSF after treatment was Borrelia negative. When specifically questioned the patient denied contact with deer nor did he remember having been bitten by ticks.

### DISCUSSION

Bilateral sixth nerve palsy with an aseptic meningitis is rarely seen in neuroborreliosis.The diagnosis in this patient is highly likely in view of the clinical picture, the excellent response to treatment and the confirmation by western blot.

There have been an increasing number of reports of Borreliosis in the United Kingdom but few outside Hampshire[1] and Scotland[2]. The disease may be under-reported. There has been only one report of a case in Wales[3],but no mention was made of where the infection may have been contracted. The preponderance of deer in the forests in this region make it likely that there is a large reservoir of the spirochaete. It would be interesting if studies on tick populations were done in the numerous recreation parks here[4].

### REFERENCES

1. Williams D., Rolles C. J., White J. E. (1986). "Lyme disease in a Hampshire child: medical curiosity or beginning of an epidemic?" *Br Med J*: **292**:1560-61.
2. Ho-Yen-D, Bennet A. J., Chisholm S., Deacon A. G., (1990). "Lyme disease in the highlands" *Scott Med J*: **Dec. [6]**: 168-70.
3. Pal G. S., Baker J. T., Humphrey P. R. D., (August 1987 [8]). "Lyme disease presenting as recurrent acute meningitis" *Br Med J*: Vol 295:367.
4. Guy E. C., Farquhar R. G. (1991). "Borrelia burgdoferi in urban parks". *Lancet*: **July [27]**: Vol 339.

